# Moderate‐Severe Thrombocytopenia Portends Poor Outcomes in Multiple Myeloma

**DOI:** 10.1002/jha2.70153

**Published:** 2025-11-20

**Authors:** Grace M. Ferri, Cenk Yildirim, Nhan V. Do, Mary T. Brophy, Nikhil C. Munshi, Camille V. Edwards, Nathanael R. Fillmore

**Affiliations:** ^1^ Department of Medicine Section of General Internal Medicine Boston Medical Center Boston Massachusetts USA; ^2^ Boston Cooperative Studies Program Informatics Center Massachusetts Veterans Epidemiology Research and Information Center (MAVERIC) Boston Massachusetts USA; ^3^ Veterans Affairs Boston Healthcare System Boston Massachusetts USA; ^4^ Section of Hematology/Oncology Boston Medical Center Boston Massachusetts USA; ^5^ Jerome Lipper Multiple Myeloma Center Dana‐Farber Cancer Institute Harvard Medical School Boston Massachusetts USA

**Keywords:** bone marrow, multiple myeloma, platelets, prognostication, tumor microenvironment

## Abstract

**Background:**

Malignant plasma cells in multiple myeloma (MM) reprogram the bone marrow microenvironment to support tumor expansion. This myeloma cell–hematopoietic stem cell interaction leads to fewer hematopoietic stem cells in the bone marrow and altered differentiation of megakaryocytes, which can contribute to MM disease‐related thrombocytopenia. Given the development of novel therapies for MM and the need for biomarkers reflecting the bone marrow microenvironment, we evaluated peripheral blood platelet count at diagnosis and during treatment of MM.

**Methods:**

We retrospectively evaluated 14,313 patients diagnosed with MM between 2000 and 2019 at Veterans Administration hospitals using platelet count obtained closest to diagnosis and up to 2.5 years thereafter. Patients were stratified into four categories:  moderate‐severe thrombocytopenia, mild thrombocytopenia, normal platelets, and thrombocytosis (< 100, 100–149, 150–349, and ≥ 350 per microliter, respectively).

**Results:**

Thrombocytopenia, present in 25% of patients at diagnosis, corresponded to inferior overall survival (OS). During follow‐up, persistent or new thrombocytopenia was also associated with inferior OS. Moreover, the negative prognostication afforded by baseline moderate–severe thrombocytopenia remained despite standard therapies (hazard ratio [HR] 1.83; 95% confidence interval [CI] 1.70–1.97) and stem cell transplant (HR 1.41; 95% CI 1.34–1.48).

**Conclusion:**

Our findings support the use of platelet count in MM as an easily accessible prognostic marker.

**Trial Registration:**

The authors have confirmed clinical trial registration is not needed for this submission.

## 1 Introduction

Malignant plasma cells in multiple myeloma (MM) reprogram the bone marrow (BM) microenvironment to support tumor expansion. In this transformative process, myeloma cells engage with hematopoietic stem cells to avoid apoptosis [1]. The myeloma cell–hematopoietic stem cell interaction results in changes to the BM microenvironment, decreased hematopoietic stem cells in the BM, and dysregulated differentiation of megakaryocytes, which can contribute to MM disease‐related thrombocytopenia [2].

Prior studies at the molecular level have shown that megakaryocytes and myeloma cells have a symbiotic relationship, where megakaryocytes in the BM microenvironment support myeloma growth and MM cells modify megakaryocyte differentiation [3]. The role of megakaryocytes in supporting the BM niche is thought to be mediated by the production of growth factors and cytokines [4]. When the BM niche is damaged, megakaryocytes express megakaryocyte‐derived factors to drive BM restoration and reconstitution [5, 6]. We previously examined absolute monocyte and lymphocyte counts as surrogates for the state of the BM milieu in MM and found that both absolute monocyte and lymphocyte counts were associated with overall survival (OS) in MM at diagnosis and follow up [7, 8]. Since changes in the BM milieu are associated with OS in MM, megakaryocytes support the BM niche and myeloma cell growth, and platelets are the downstream differentiation product of megakaryocytes, we hypothesized that peripheral blood platelet count may similarly be employed as a surrogate marker for megakaryocytic precursors in the BM microenvironment [7, 8, 9, 10]. In light of the development of novel therapies for MM and need for prognostic markers that reflect the underlying BM microenvironment, we sought to evaluate peripheral blood platelet count as a dynamic prognostic biomarker in MM.

## 2 Methods

### 2.1 Patients

This retrospective cohort study was conducted with data supplied from the Veterans Affairs (VA) Corporate Data Warehouse (CDW); the CDW collects and combines electronic health record and cancer registry information from VA facilities across the nation. All patients diagnosed with MM between 1 January 2000 and 31 December 2019 were included, where identification was performed using the 9th and 10th editions of the international classification of diseases (ICD) codes for MM. All included patients were required to have (a) at least three visits from separate days with an MM ICD code; (b) therapy with at least one drug for MM (not including corticosteroids) on or after the date of the first visit with an MM ICD code; and (c) at least one platelet measurement on or within 90 days before diagnosis date; diagnosis date was uniformly considered to be the date when the first MM treatment was administered. Due to platelet sequestration and decreased thrombopoietin production in cirrhosis, we excluded any patients with chronic liver disease. Patients with other hematologic malignancies (including acute and chronic leukemias, aplastic anemia, myelodysplastic syndrome, hairy cell leukemia, or myeloproliferative neoplasms diagnosed before MM diagnosis) were excluded, as these conditions and their associated therapies could cause or contribute to an abnormal platelet count; accordingly, if other hematologic malignancies arose during follow‐up, we stopped observing and censored follow‐up data for these patients starting at the diagnosis date for the other hematologic malignancy. Time from baseline to follow‐up was defined as time from diagnosis until development of any of the following: other hematologic malignancy, death, truncation date (15 years after MM diagnosis), or the study end date. Follow‐up analyses were then performed in 6‐month increments from diagnosis date (0 years) to 2.5 years through 31 December 2019.

### 2.2 Study Variables and Assessments

Variables were collected during baseline (diagnosis) and follow up: 6 months, 1 year, 1.5 years, 2 years, and 2.5 years. Baseline data included diagnosis date, age, sex, race, ethnicity, serum LDH, beta 2 microglobulin (β2m), serum albumin, serum creatinine, Charlson comorbidity index (CCI), era of treatment, and occurrence of thromboembolic or bleeding events within 3 years of baseline data acquisition. Follow‐up variables included exposure to standard combination therapy with lenalidomide and bortezomib administered during and after 2012. Age (continuous variable), sex, race (black, white, other), and ethnicity were characterized according to the data classification in the CDW. Staging was calculated using baseline data (with closest serum albumin and β2m measurements within 90 days prior to the index date) and classified according to the ISS; ISS is a known predictor of OS at MM diagnosis. Time of treatment fell into three eras: < 2007, 2007–2011 and ≥ 2012. CCI was designated using ICD‐10 codes.

We obtained baseline and follow‐up platelet count among patients, where platelet count was computed using an automated or manual differential cell count. We used the platelet count obtained within 90 days before and closest to MM diagnosis and then at 6‐month intervals until 2.5 years post‐diagnosis. The cut‐off of 2.5 years was established based on the median OS of our cohort. Platelet count at the time point of interest was used to stratify patients in platelet categories for the final analysis. Patients were stratified into four categories: moderate–severe thrombocytopenia, mild thrombocytopenia, normal platelets, and thrombocytosis (< 100, 100–149, 150–349, and ≥ 350 per microliter, respectively) based on institutional reference ranges. To prevent treatment‐related changes from interfering with the accuracy of obtained platelet counts, any platelet count acquired within 7 days of an abnormal neutrophil count was excluded.

### 2.3 Statistical Analysis

We used descriptive statistics (means, medians, and interquartile ranges [IQRs]) to summarize patient demographic and baseline characteristics. When comparing baseline characteristics between the four platelet categories, we used the nonparametric Kruskal–Wallis test (for continuous variables) and the *χ* ^2^ test (for categorical variables). OS was the primary outcome estimated by Kaplan–Meier method and log‐rank tests. Survival analysis at each time increment featured those patients with available laboratory data and treatment was included as a time‐varying covariate. Cox proportional hazard models (adjusted for age, race, CCI, smoking, ISS, LDH, creatinine, and era of treatment) were used to obtain HR. Additional analyses were performed at follow‐up time points. Multiple imputation by chained equations method was used to impute missing covariates. Statistical analyses were performed using R (version 4.4.1 software package). Statistical tests were two‐sided and *p* < 0.05 was considered statistically significant.

### 2.4 Study Oversight

The VA Boston Research and Development committee approved this study as exempt prior to data collection and analysis. Need for informed consent was waived given the use of existing data, per the Common Rule.

### 2.5 Data Sharing

Individual‐level data within this study is available to researchers with VA regulatory approval in accordance with VA policy.

## 3 Results

Between 1 January 2000 and 31 December 2019, 14,313 patients with newly diagnosed MM at nationwide VA medical centers met criteria for inclusion. Demographic and clinical features are highlighted in Table 1. The median age at diagnosis was 69.8 years (IQR 63.0–77.0 years), where 98% of patients were male. Thrombocytopenia (including mild and moderate–severe) was present in 25% of patients at MM diagnosis (3548 of 14,313 patients), where 17% and 7% of patients had severely mild and moderate–severe thrombocytopenia, respectively. Moreover, 27% of patients age > 65 had thrombocytopenia at MM diagnosis compared to 20% of patients age ≤ 65 at MM diagnosis. Approximately 78% of the 7253 evaluable patients in the overall cohort met the criteria for ISS Stages 2 and 3 MM. Patients with thrombocytosis were more likely to experience venous thromboembolic events (odds ratio [OR] 1.62, 95% CI 1.29–2.02) relative to those with normal platelet counts (OR 1.33, 95% CI 1.17–1.52). No obvious differences in rates of bleeding events within 3 years of diagnosis were observed among the four platelet classes (Table 1).

**TABLE 1:** Demographics and clinical characteristics of patients with MM at diagnosis included in study (*n* = 14,313)

	**Platelet Count (x 10^3^/mm^3^)**					
**Overall**	**Overall**	**Moderate–severe thrombocytopenia (< 100)**	**Mild thrombocytopenia (100–149)**	**Normal (150–349)**	**Thrombocytosis (≥ 350)**	***p* value**
**Number of patients, *n* (%)**	14,313 (100)	1060 (7.4)	2488 (17.4)	9906 (69.2)	859 (6.0)	
**Age at diagnosis, median [IQR]**	69.8 [63.0, 77.0]	70.6 [63.1, 78.2]	71.9 [65.5, 78.2]	69.6 [62.7, 76.6]	66.1 [59.5, 73.0]	< 0.001
**Years of follow up, median [IQR]**	2.3 [0.9, 4.5]	1.1 [0.3, 2.7]	2.1 [0.8, 4.2]	2.5 [1.1, 4.8]	2.2 [0.8, 4.6]	< 0.001
**Age ≥ 65 at diagnosis, *n* (%)**	9738 (68)	731 (7.5)	1904 (20)	6639 (68)	464 (4.8)	< 0.001
**Male gender, *n* (%)**	13,977 (97.7)	1038 (7.4)	2446 (17.5)	9667 (69.2)	826 (5.9)	0.003
**Race, *n* (%)**						< 0.001
** White**	8477 (59.2)	626 (7.4)	1521 (17.9)	5865 (69.2)	465 (5.5)	
** Black**	3526 (24.6)	224 (6.4)	581 (16.5)	2480 (70.3)	241 (6.8)	
**Ethnicity, *n* (%)**						< 0.001
** Hispanic or Latino**	694 (4.8)	38 (5.5)	104 (15.0)	513 (73.9)	39 (5.6)	
** Not Hispanic or Latino**	11,866 (82.9)	859 (7.2)	2058 (17.3)	8249 (69.5)	700 (5.9)	
**MM diagnosis date, *n* (%)**						< 0.001
** < 2007**	4039 (28.2)	287 (7.1)	546 (13.5)	2872 (71.1)	334 (8.3)	
** ≥ 2007 to < 2012**	3509 (24.5)	287 (8.2)	617 (17.6)	2407 (68.6)	198 (5.6)	
** ≥ 2012**	6765 (47.3)	486 (7.2)	1325 (19.6)	4627 (68.4)	327 (4.8)	
**Transplant Exposure, *n* (%)**	1845 (12.9)	81 (4.4)	252 (13.7)	1394 (75.6)	118 (6.4)	< 0.001
**No transplant, *n* (%)**	12,468 (87.1)	979 (7.9)	2236 (17.9)	8512 (68.3)	741 (5.9)	
**Transplant within 1 year, *n* (%)**	1281 (8.9)	63 (4.9)	190 (14.8)	944 (73.7)	84 (6.6)	
**Transplant after 1 year, *n* (%)**	564 (3.9)	18 (3.2)	62 (11.0)	450 (79.8)	34 (6.0)	
**Median platelet count (x10^3^/mm^3^) [IQR])**	197 [150, 251]	77 [57, 90]	128 [115, 139]	215 [182, 256]	400 [372, 449]	< 0.001
**Distribution of ISS stages across the four PLT groups, *n* (%)** ^*^						< 0.001
** Stage 1**	1619 (11.3)	46 (4.3)	204 (8.2)	1270 (12.8)	99 (11.5)	
** Stage 2**	2854 (19.9)	153 (14.4)	406 (16.3)	2071 (20.9)	224 (26.1)	
** Stage 3**	2780 (19.4)	292 (27.5)	579 (23.3)	1755 (17.7)	154 (17.9)	
**Smoking status (%)**						< 0.001
** Never**	2796 (19.5)	228 (8.2)	514 (18.4)	1911 (68.3)	143 (5.1)	
** Current/former**	10,341 (72.2)	714 (6.9)	1760 (17.0)	7242 (70.0)	625 (6.0)	
**Charlson Comorbidity Index (CCI score)** ^*^						0.001
** No comorbidities**	517 (3.6)	39 (3.7)	98 (3.9)	351 (3.5)	29 (3.4)	
** Mild (1‐2)**	7546 (52.7)	522 (49.2)	1265 (50.8)	5327 (53.8)	432 (50.3)	
** Moderate (3‐4)**	4225 (29.5)	323 (30.5)	720 (28.9)	2899 (29.3)	283 (32.9)	
** Severe (> 5)**	1861 (13.0)	159 (15.0)	366 (14.7)	1226 (12.4)	110 (12.8)	
**Venous thromboembolic events, *n* (%)**	1613 (11.3)	89 (5.5)	235 (14.6)	1169 (72.5)	120 (7.4)	< 0.001
**Overall bleeding, *n* (%)**	3471 (24.3)	206 (5.9)	640 (18.4)	2424 (69.8)	201 (5.8)	0.001
**Intracranial bleeding, *n* (%)**	572 (4.0)	38 (6.6)	114 (19.9)	381 (66.6)	39 (6.8)	0.268
**Gastrointestinal bleeding, *n* (%)**	3266 (22.8)	189 (5.8)	600 (18.4)	2292 (70.2)	185 (5.7)	< 0.001
**Deletion 17p**						< 0.001
** Absent, *n* (%)**	1909 (13.3)	130 (6.8)	321 (16.8)	1369 (71.7)	89 (4.7)	
** Present, *n* (%)**	189 (1.3)	28 (14.8)	47 (24.9)	108 (57.1)	6 (3.2)	
**Serum albumin** ^*^						< 0.001
** Albumin < 3.5 g/dL (%)**	6493 (45.4)	581 (54.8)	1128 (45.3)	4289 (43.3)	495 (57.6)	
** Albumin, median [IQR] (g/dL)**	3.50 [3.00, 3.90]	3.30 [2.70, 3.80]	3.50 [3.00, 3.90]	3.50 [3.07, 3.90]	3.20 [2.70, 3.80]	< 0.001
**β2m**						< 0.001
** β2m > 3.5 mcg/mL (%)**	4629 (32.3)	406 (8.8)	874 (18.9)	3071 (66.3)	278 (6.0)	
** Creatinine, median [IQR] (mg/dL)**	1.21 [1.00, 1.80]	1.40 [1.10, 2.30]	1.30 [1.00, 1.92]	1.20 [1.00, 1.70]	1.16 [0.90, 1.70]	< 0.001
**Serum LDH**						< 0.001
** LDH >243 U/L, *n* (%)**	1484 (10.4)	213 (14.4)	259 (17.5)	896 (60.4)	116 (7.8)	

* Denotes column percentages.

### 3.1 Inferior OS in Patients With Moderate–Severe Thrombocytopenia at Diagnosis

**FIGURE 1:**
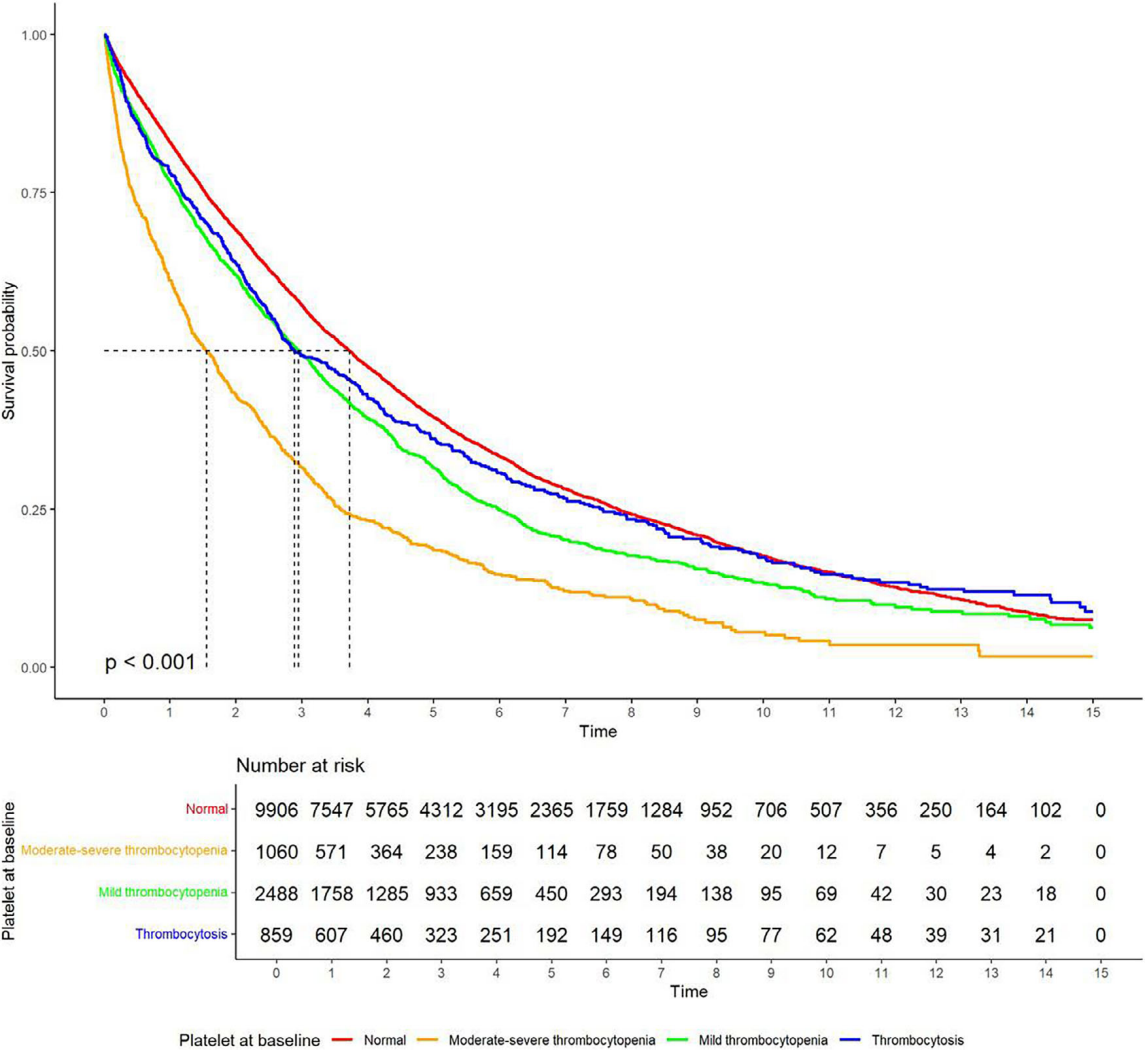
Kaplan–Meier curves of OS based on platelet count at diagnosis of MM.

Patients with thrombocytopenia at diagnosis had inferior OS compared to patients with normal platelet count or thrombocytosis. Median OS of patients with moderate–severe thrombocytopenia, mild thrombocytopenia, normal platelets, and thrombocytosis at diagnosis was 1.6, 3.0, 3.7, and 2.9 years (*p* <.001), respectively (Figure 1).

**FIGURE 2:**
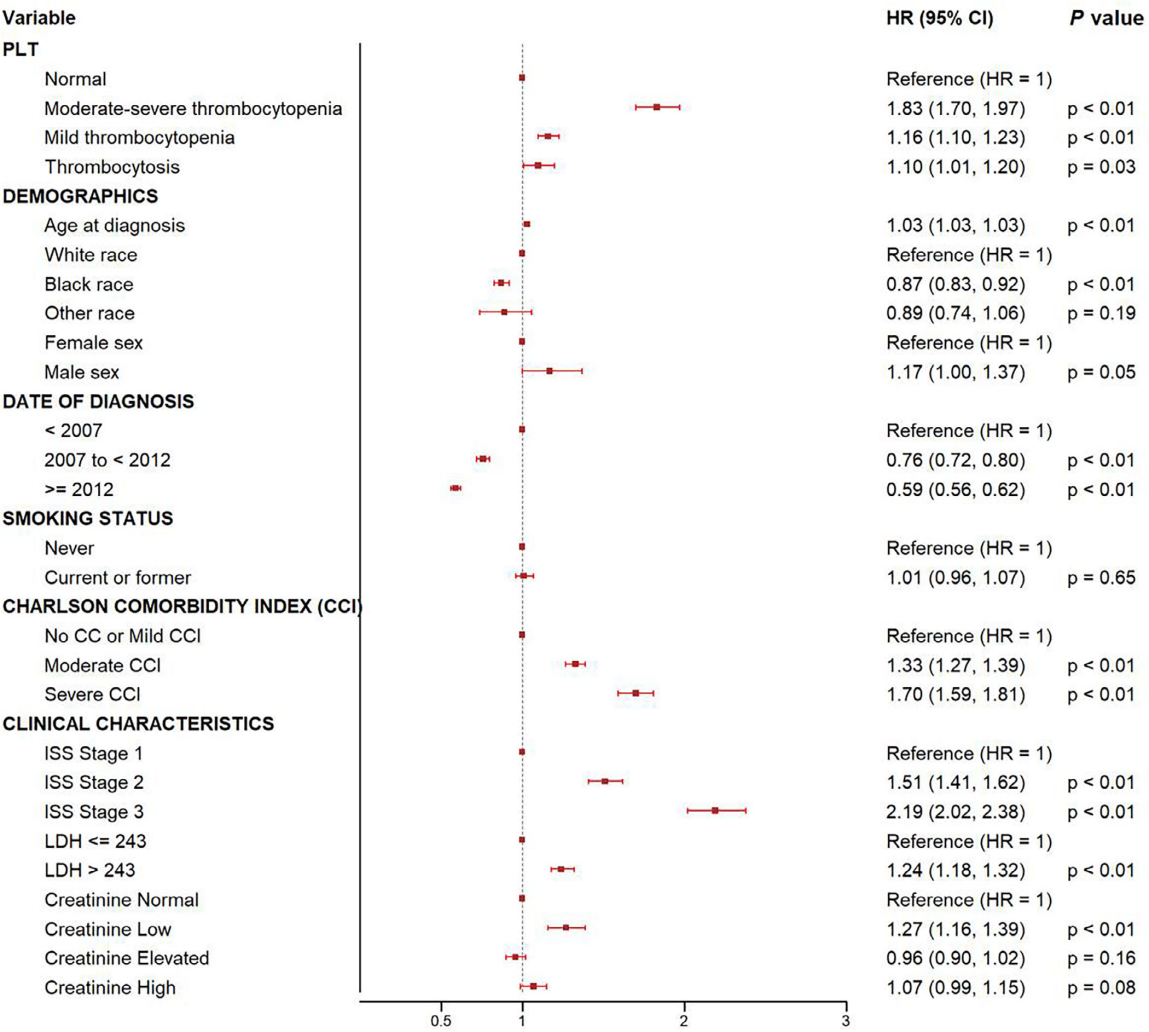
Multivariable analysis of the main prognostic factors for OS at diagnosis. CI, confidence interval.

**FIGURE 3:**
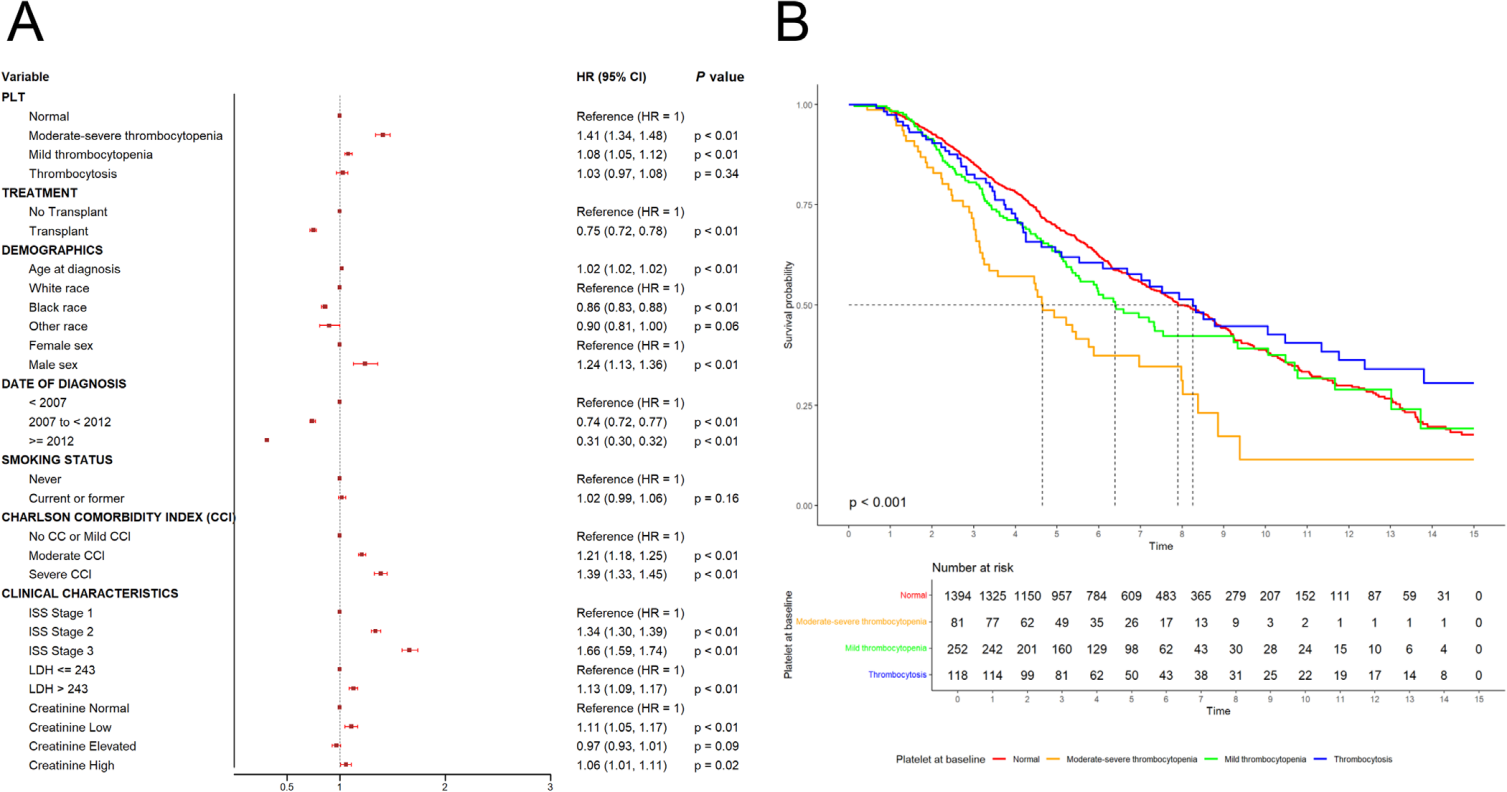
(A) Multivariable analysis of the main prognostic factors for OS at diagnosis of MM using transplant as time‐varying exposure. (B) Kaplan–Meier curves of OS by platelet count among patients receiving stem cell transplant.

The negative prognostication accompanying baseline thrombocytopenia persisted despite standard therapies such as lenalidomide and bortezomib. Patients with moderate–severe thrombocytopenia had the worst outcomes when treated with standard therapy (HR 1.83; 95% CI: 1.70–1.97) relative to those with mild thrombocytopenia or thrombocytosis (HR 1.16; 95% CI: 1.10–1.23 and HR 1.10; 95% CI 1.01–1.20 for mild thrombocytopenia and thrombocytosis, respectively; Figure 2). Moreover, although stem cell transplant is associated with improved mortality independent of baseline platelet count (HR 0.75, CI: 0.72–0.78), those with baseline moderate–severe thrombocytopenia continued to have the worst survival (HR 1.41; 95% CI: 1.34–1.48; OS 4.6 years; Figure 3A,B) after transplant relative to their counterparts (OS: 7.9 years, 6.4 years, 4.6, and 8.3 years for moderate–severe thrombocytopenia, normal platelets, mild thrombocytopenia, and thrombocytosis, respectively; Figure 3B).

### 3.2 Moderate–Severe Thrombocytopenia During Follow‐Up Is Associated With Poor OS

**FIGURE 4:**
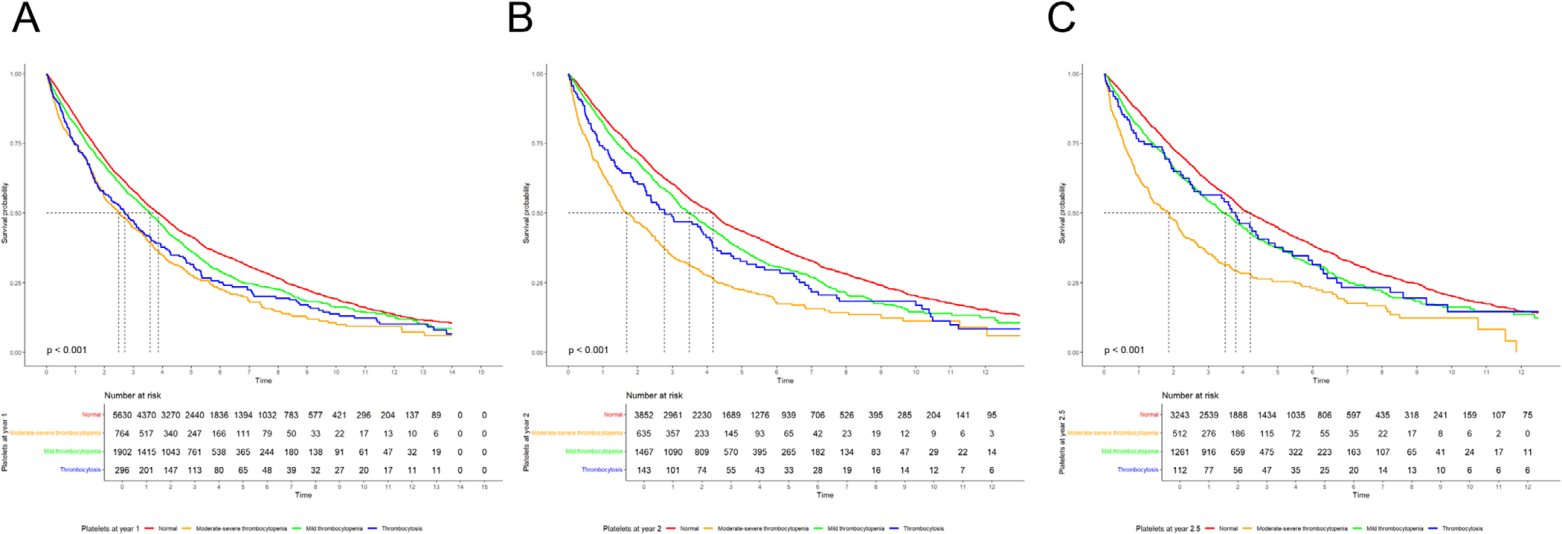
Kaplan–Meier curves of OS based on platelet count (A) 1 year, (B) 2 years, and (C) 2.5 years after MM diagnosis.

We evaluated platelet count at follow‐up time points after induction therapy. Appearance or persistence of moderate–severe thrombocytopenia during follow‐up was associated with poor outcomes. In particular, moderate–severe thrombocytopenia at 1, 2, and 2.5 years after MM diagnosis was associated with significantly lower OS (Figure 4A–C). Median OS at 1 year was 2.5, 3.6, 3.9, and 2.7 (*p* < 0.001), median OS at 2 years was 1.7, 3.5, 4.2, and 2.8 (*p* < 0.001), and median OS at 2.5 years was 1.9, 3.5, 4.2, and 3.8 (*p* < 0.001) years among patients with moderate–severe thrombocytopenia, mild thrombocytopenia, normal platelet count, or thrombocytosis, respectively.

We further evaluated whether developing changes in platelet count at MM follow‐up was predictive of outcome. Among those who had moderate–severe thrombocytopenia at baseline, improvement in platelet count to normal levels did not translate to any statistically significant changes in OS over a 2.5‐year period (Figure S1). Among those with normal platelets at baseline, the development of moderate–severe thrombocytopenia was associated with the worst median OS at all time points from 0.5 to 2.5 years (Figure S3).

### 3.3 Moderate–Severe Thrombocytopenia is Independently Associated With Adverse Outcomes in the Era of Novel Therapies

**FIGURE 5:**
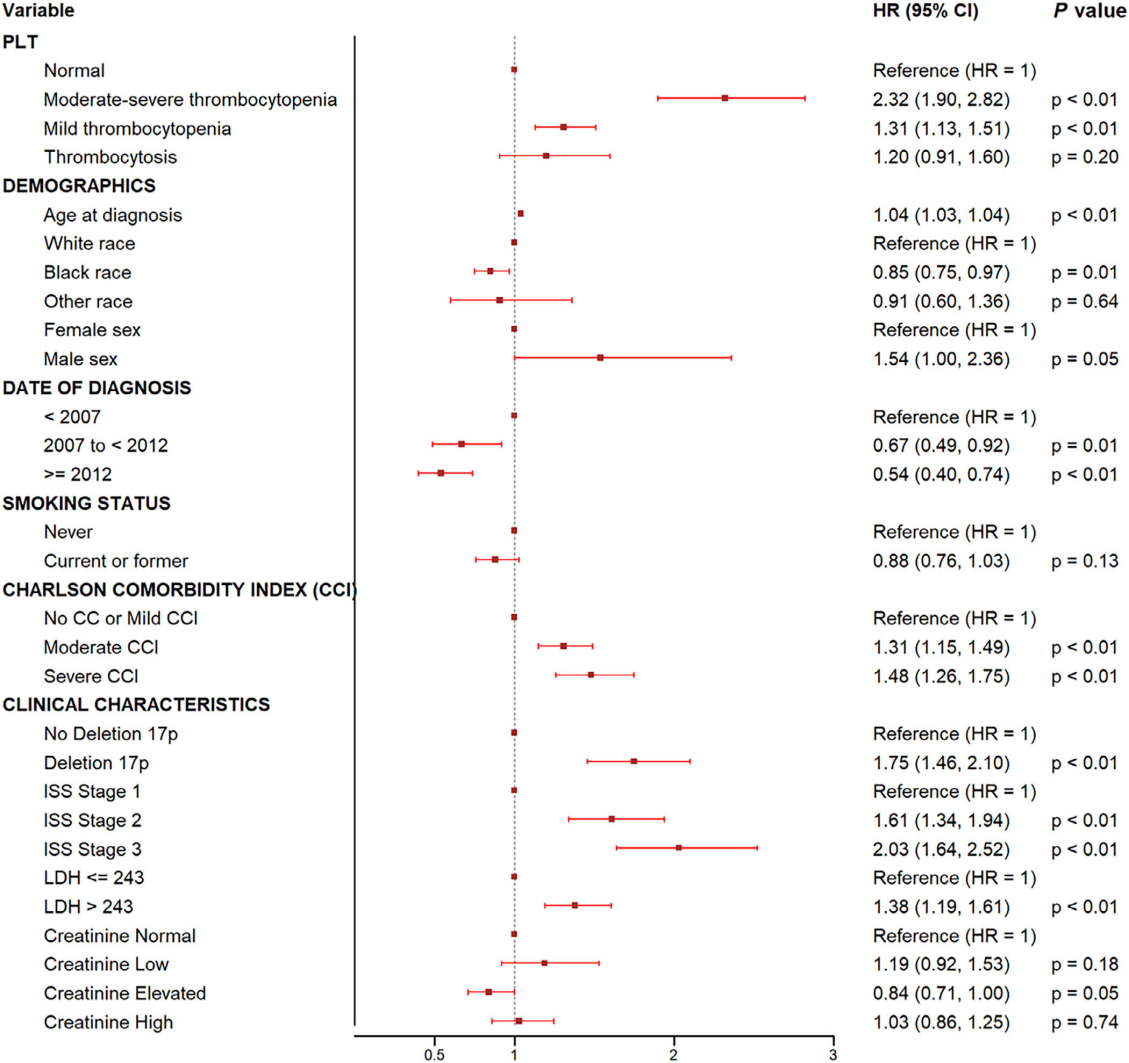
Multivariable analysis of the main prognostic factors for OS at diagnosis of MM among patients in whom deletion 17p data were available. CI, confidence interval.

We then assessed if the prognostication afforded by platelet count would persist with the use of novel agents. We compared the impact of platelet count on the OS of patients treated before or after 2012 when novel therapeutics were utilized universally across the VA healthcare system. As anticipated, the median OS improved across all platelet groups in patients with MM diagnosed after 2012. Median OS was 1.3, 2.4, 3.2, and 2.6 years (*p* < 0.001) among patients with moderate–severe thrombocytopenia, mild thrombocytopenia, normal platelet count, or thrombocytosis diagnosed before 2012 compared to those treated after 2012 with median OS 1.9, 3.6, 4.7, and 4.8 years (*p* < 0.001) (Figure S5). Moreover, multivariable analyses demonstrated that thrombocytopenia is an independent prognostic factor predicting poor outcomes independent of validated predictors of OS at MM diagnosis including age, ISS stage, and LDH (HR 1.83; 95% CI: 1.70–1.97) for moderate–severe thrombocytopenia and HR 1.16; 95% CI: 1.10–1.23 for mild thrombocytopenia) at diagnosis (Figure 2). Interestingly, patient's age < 65 with moderate–severe thrombocytopenia were more likely to suffer poor MM outcomes than their counterpart's age > 65 (HR 2.05; 95% CI: 1.79–2.35 vs. HR 1.74; 95% CI: 1.59–1.90). Among those in whom deletion 17p data were available at diagnosis, thrombocytopenia remained predictive of even worse OS (HR 2.32; 95% CI: 1.90–2.82 for moderate–severe thrombocytopenia and HR 1.31; 95% CI: 1.13–1.51 for mild thrombocytopenia) (Figure 5).

## 4 Discussion

Building on prior reports suggesting that peripheral blood markers could reflect the MM BM microenvironment and smaller studies correlating thrombocytopenia in MM with mortality risk, we have demonstrated in this work that platelet count at diagnosis and follow‐up predicts OS in MM [2, 7]. For patients with normal platelet count at diagnosis, developing moderate–severe thrombocytopenia after diagnosis similarly portends inferior survival compared to their counterparts maintaining a normal platelet count. Platelet count also confers adverse outcomes independent of validated prognostic markers (i.e., prediction tools such as ISS stage and CCI). Our cohort represents the largest study examining this phenomenon during diagnosis, follow up, and exposure to real‐world treatment modalities including HSCT.

In our study, patients with moderate–severe thrombocytopenia at diagnosis experienced the lowest survival rates. We posit that thrombocytopenia may be a surrogate marker of myelomatous involvement of the tumor microenvironment, where lower platelets may theoretically indicate increased plasma cell involvement in the BM. The particularly poor OS seen among those with moderate–severe thrombocytopenia developing thrombocytosis or vice versa may reflect clonal expansion of myeloma cells expressing IL‐6, which mediates platelet production [11].

Furthermore, decreased OS persisted over time despite receiving combination therapy with lenalidomide and bortezomib and HSCT. The continued poor OS among patients with baseline moderate‐severe thrombocytopenia receiving HSCT raises concern for unaddressed molecular or metabolic factors within the BM microenvironment predisposing to poor BM engraftment [2]. Among patients who have undergone HSCT, persistent thrombocytopenia has been associated with increased mortality [12]. Potential mechanisms underlying this phenomenon include but are not limited to: inability to mount a sufficient megakaryocyte response, delayed platelet engraftment, acute graft‐versus‐host disease, cytomegalovirus infection, and immune thrombocytopenia [13, 14, 15, 16, 17]. Prior clinical trials have studied the use of thrombopoietin receptor agonists among such patients with prolonged thrombocytopenia after HSCT and have demonstrated exceedingly poor survival despite numerical improvements in platelet counts [12, 18]. Our data suggests that HSCT, while beneficial in improving OS, does not overcome the negative prognostic impact of pre‐HSCT thrombocytopenia. We postulate that those components of the BM ecosystem predisposing to moderate–severe thrombocytopenia at diagnosis may also contribute to delayed platelet engraftment following HSCT. Translating these results into clinical practice may lead to implementing a higher target dose of CD34‐positive cells required for HSCT among those with baseline moderate–severe thrombocytopenia [19, 20, 21, 22, 23]. Standardizing higher threshold doses of CD34‐positive cells could increase probability of BM recovery and, in turn, successful and timely platelet engraftment.

Our study is limited by the predominance of male participants comprising the United States VA healthcare system. Of note, we must acknowledge that the peripheral blood platelet count may not directly and independently translate to underlying MM disease trajectory at all time points. Thrombocytopenia may be erroneous (pseudothrombocytopenia) if platelet clumping is present or multifactorial in the setting of unreported herbal substances, immune thrombocytopenia, acute viral illness, and chronic liver disease in addition to the hypothesized mechanisms mentioned above. However, none of these mechanisms, with the exception of chronic liver disease, should correlate so strongly and persistently with thrombocytopenia as does progressive MM. While agents such as bortezomib and lenalidomide prescribed during MM treatment could induce myelosuppression, any thrombocytopenia incurred would be expected to be limited to the duration of treatment and thus terminate with treatment discontinuation. Prior studies suggest that bortezomib‐induced thrombocytopenia is transient and cyclic due to a reversible impairment of megakaryocyte function rather than direct cytotoxicity to BM precursor cells [24, 25]. We cannot definitely ascribe the thrombocytopenia observed to the underlying myeloma or the therapies administered. If bortezomib or lenalidomide were held owing to dose‐limiting thrombocytopenia, patients may be more prone to disease progression. However, the inability to maintain platelet counts on these routinely used medications is more likely a reflection of the disease itself.

In conclusion, we illustrate that the peripheral blood platelet count is a readily available biomarker offering time‐varying prognostication. Based on our findings, platelet count considered at 6‐month intervals during the first 2.5 years of MM treatment should detect patients with poor prognosis. If peripheral blood markers such as platelet count were featured in time‐varying predictive models during treatment, risk‐adapted therapeutic strategies would then be possible longitudinally—independent of staging or abnormal cytogenetics at diagnosis. Furthermore, as baseline thrombocytopenia may also portend poorer outcomes with HSCT, we propose routine consideration of platelet counts in treatment schedules and peritransplant decision‐making. This study supports future efforts to validate a novel prognostic index featuring the platelet count for use in clinical practice.

## Author Contributions

G.M.F., C.Y., N.C.M., N.R.F., and C.V.E. designed research, and interpreted results. G.M.F. wrote the manuscript. C.Y. performed the research and analyzed data. All authors edited the manuscript.

## Conflicts of Interest

The views expressed in this article are those of the authors and do not necessarily reflect the position or policy of the Department of Veterans Affairs or the US government. Potential conflicts of interests include research funding from Merck (NRF) and consultancy or advisory role with Adaptive, Amgen, BMS, Celgene, Janssen, Novartis, Pfizer, Legend, and Takeda and stock for Oncopep and DCT (NCM).

## Supporting information




**Figure S1**: Moderate‐severe thrombocytopenia at baseline, KMs by Platelets at FUP.**Figure S2**: Mild Thrombocytopenia at baseline, KMs by Platelets at FUP.**Figure S3**: KM‐Normal Platelets at baseline, KMs by Platelets at FUP.**Figure S4**: Thrombocytosis at baseline, KMs by Platelets at FUP.**Figure S5**: OS based on baseline platelet count at MM diagnosis with respect to the era of diagnosis and treatment: (A) <2012 and (B) ≥2012.**Figure S6**: OS based on baseline platelet count at MM diagnosis and RVd induction therapy for patients diagnosed ≥2012.**Figure S7**: OS based on baseline platelet count as both a fixed baseline (up to year 2) and a time‐varying covariate (years 2–4).

## Data Availability

Data available upon reasonable request to corresponding author.
