# Supplementary material for: Moderate‐Severe Thrombocytopenia Portends Poor Outcomes in Multiple Myeloma
Source: EJHaem. 2025 Nov 20;6(6):e70153. doi: 10.1002/jha2.70153 (PMC12631539; doi:10.1002/jha2.70153)

Supplemental Figure 1. Moderate-severe thrombocytopenia at baseline, KMs by Platelets at FUP
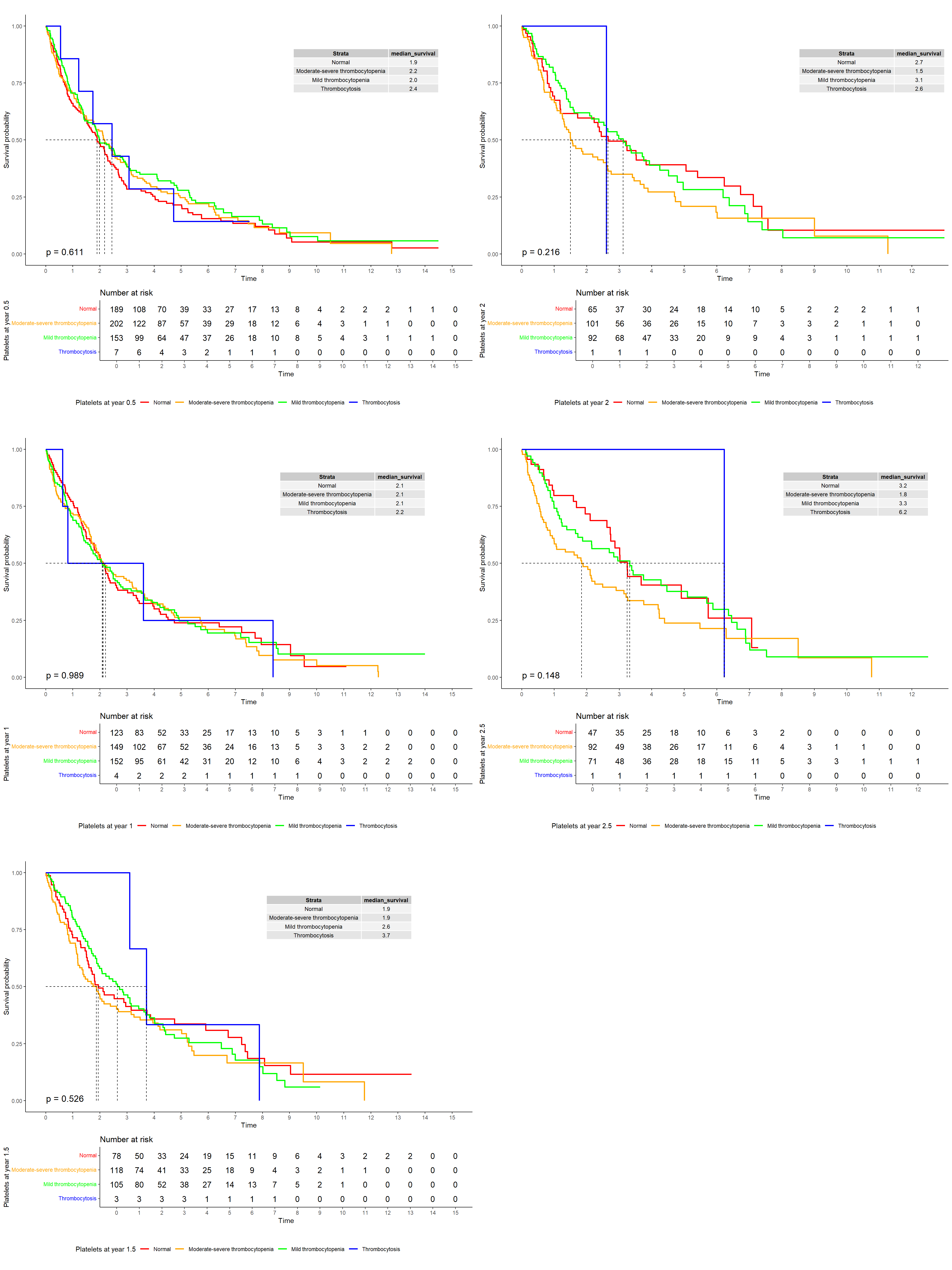


Supplemental Figure 2. Mild Thrombocytopenia at baseline, KMs by Platelets at FUP


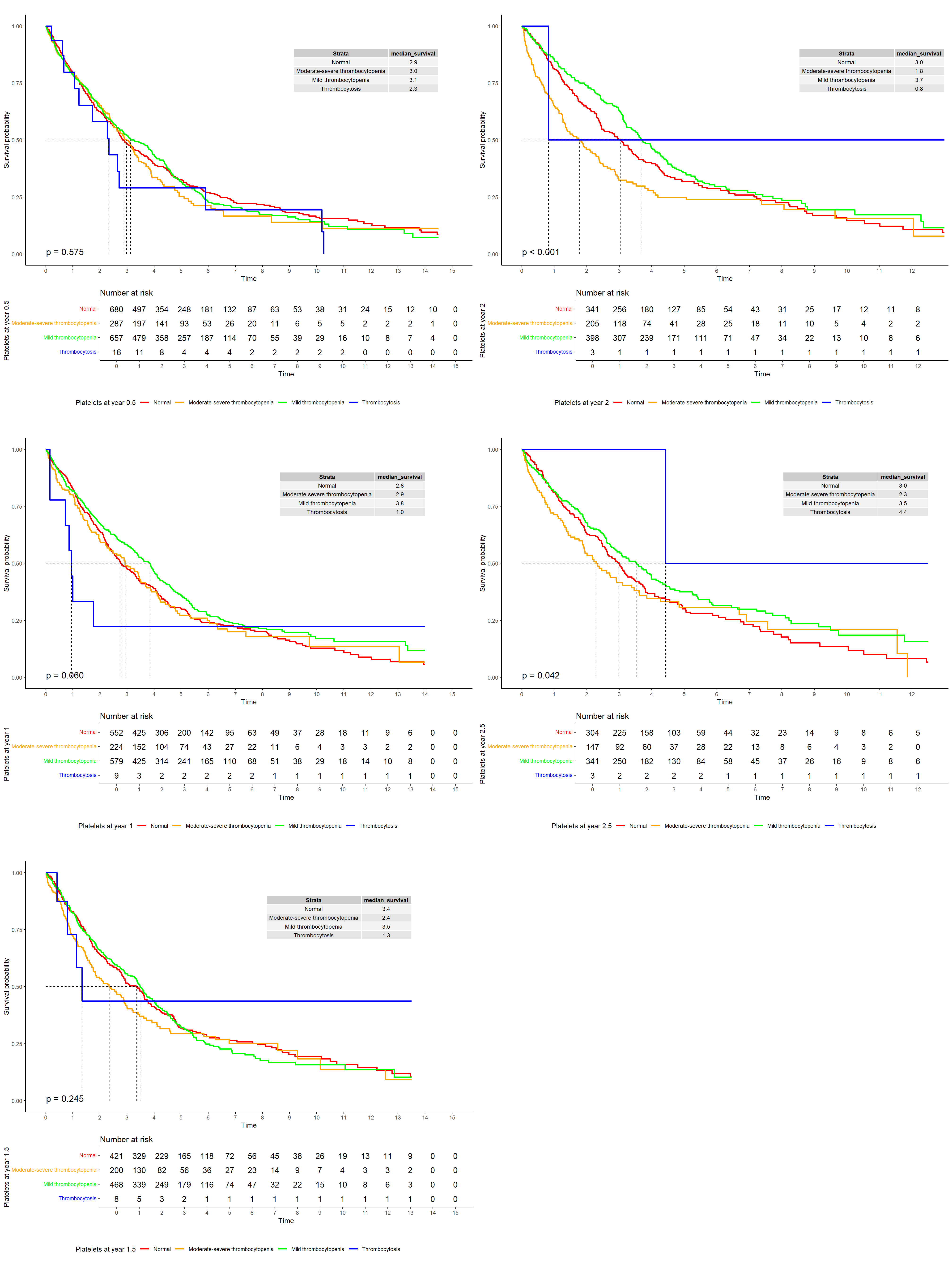


Supplement Figure 3. KM - Normal Platelets at baseline, KMs by Platelets at FUP


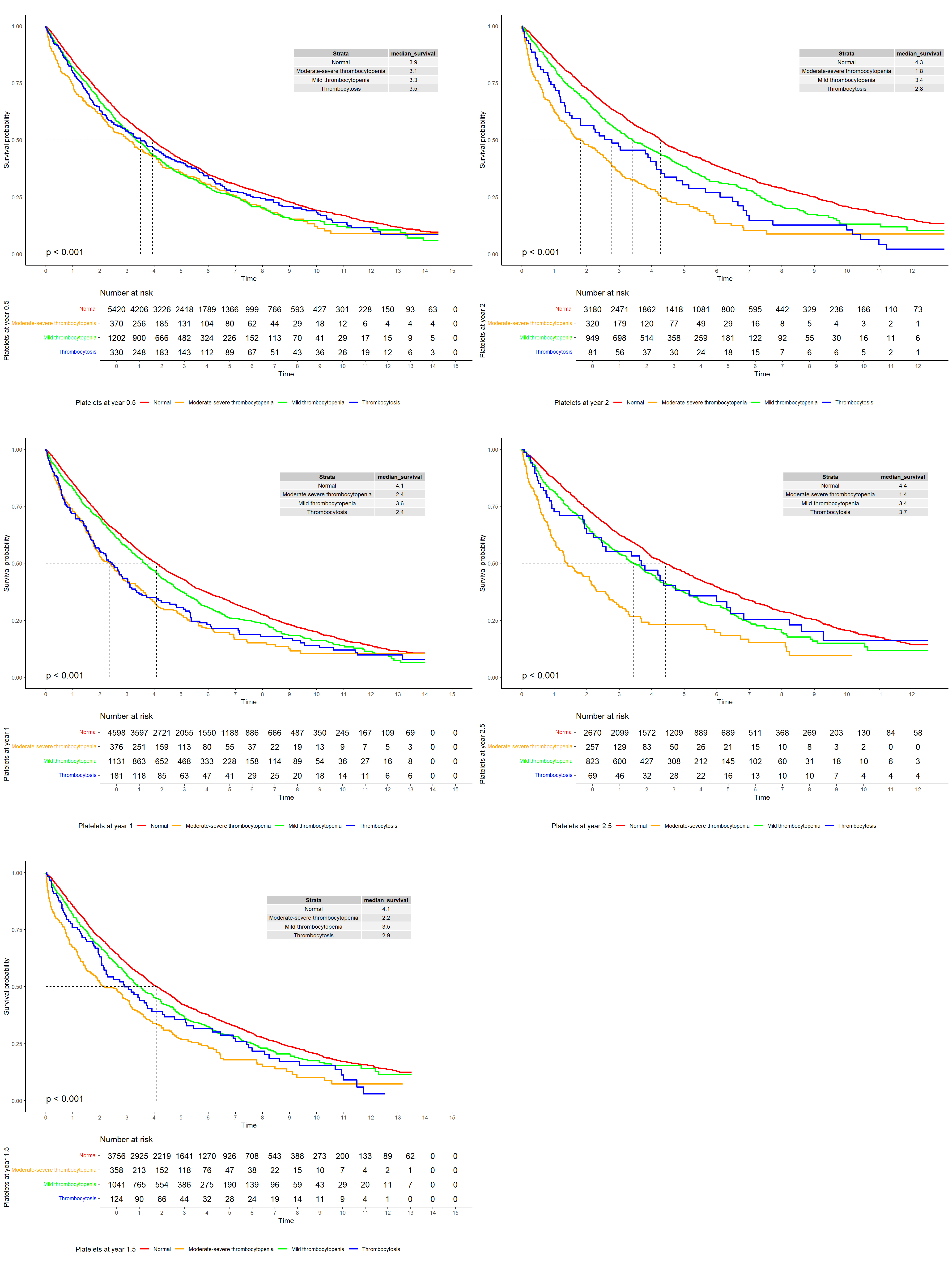


Supplemental Figure 4. Thrombocytosis at baseline, KMs by Platelets at FUP


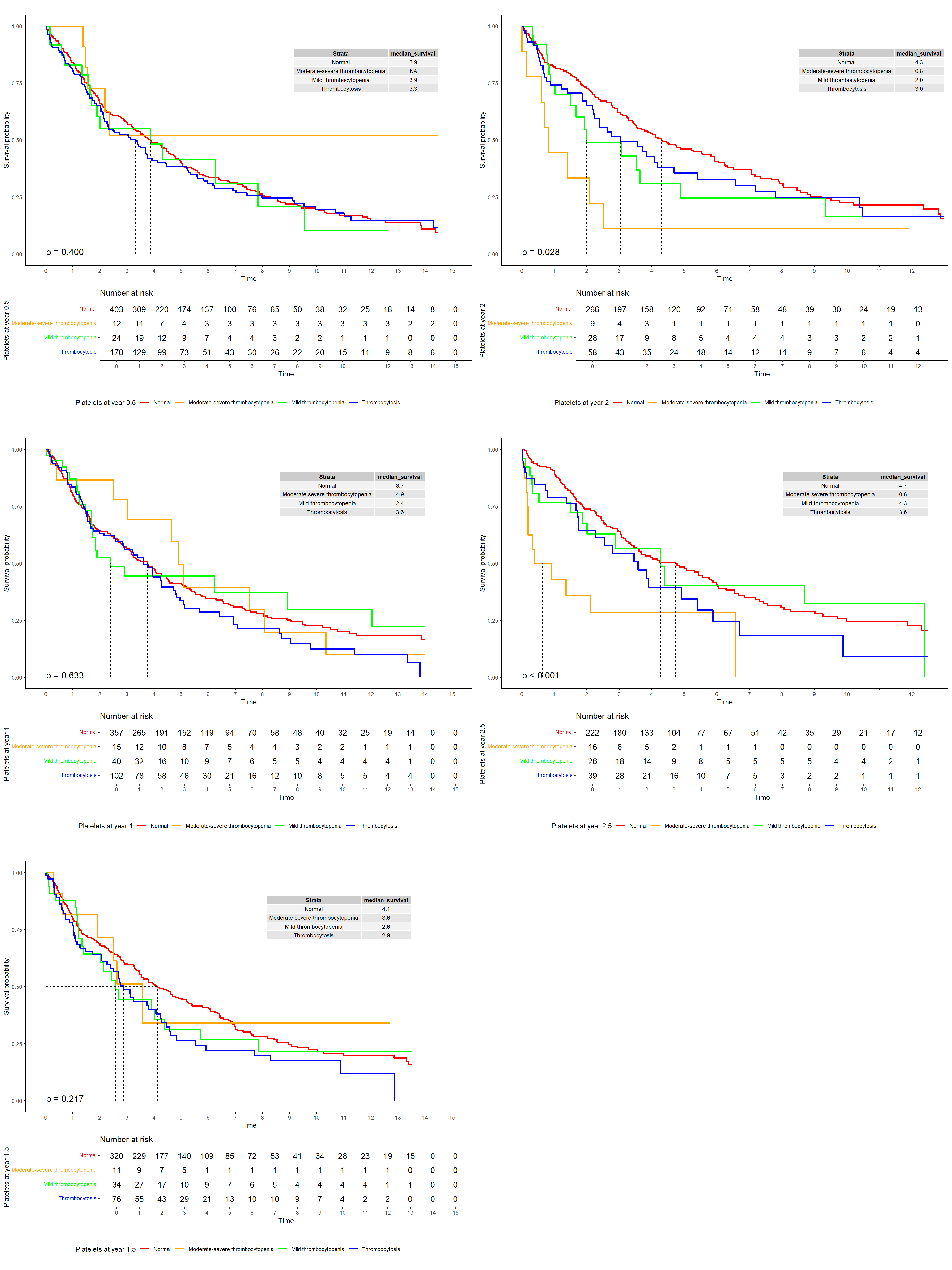


Supplemental Figure 5. OS based on baseline platelet count at MM diagnosis with respect to the era of diagnosis and treatment: (A) <2012 and (B) ≥2012.

(A) <2012


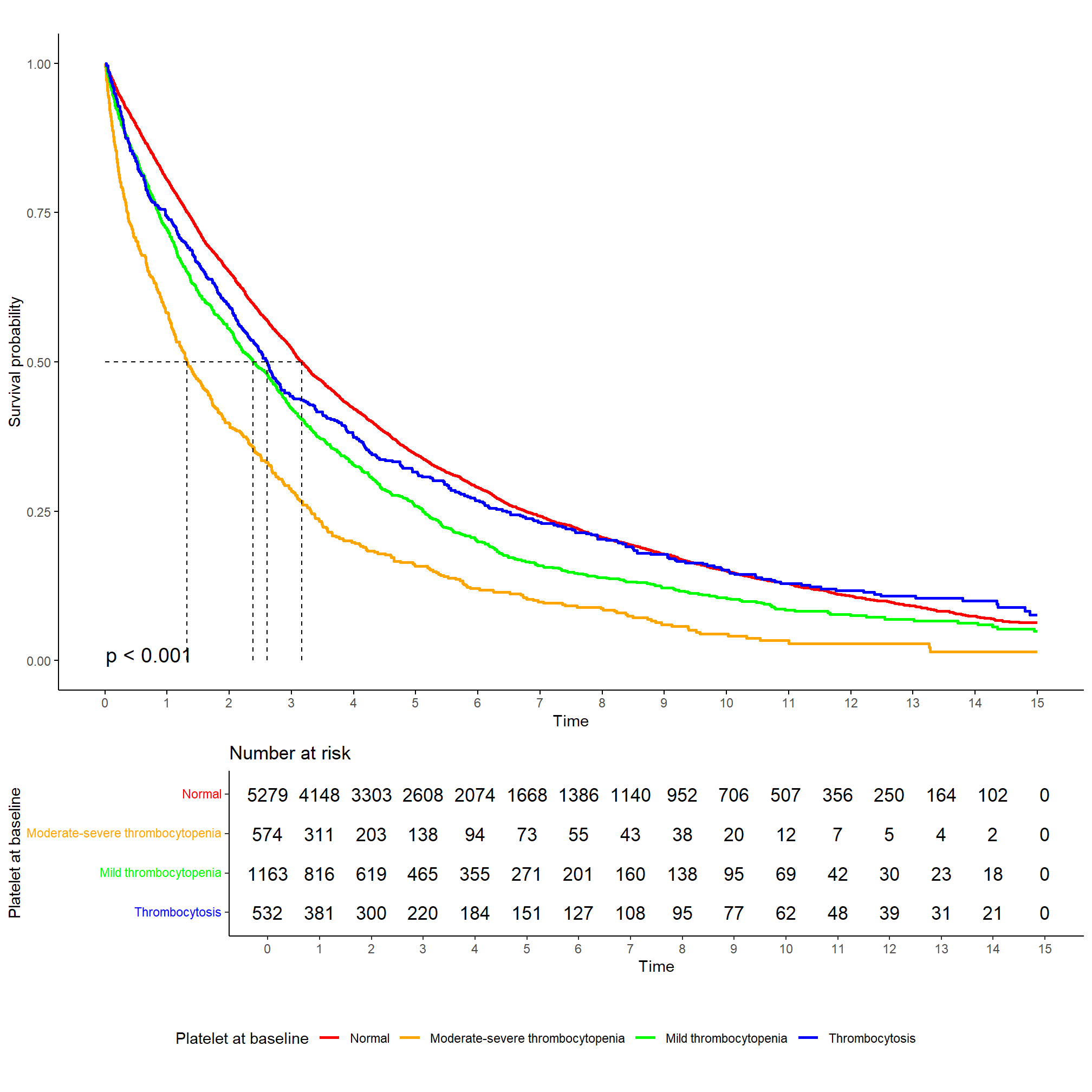


(B) ≥2012


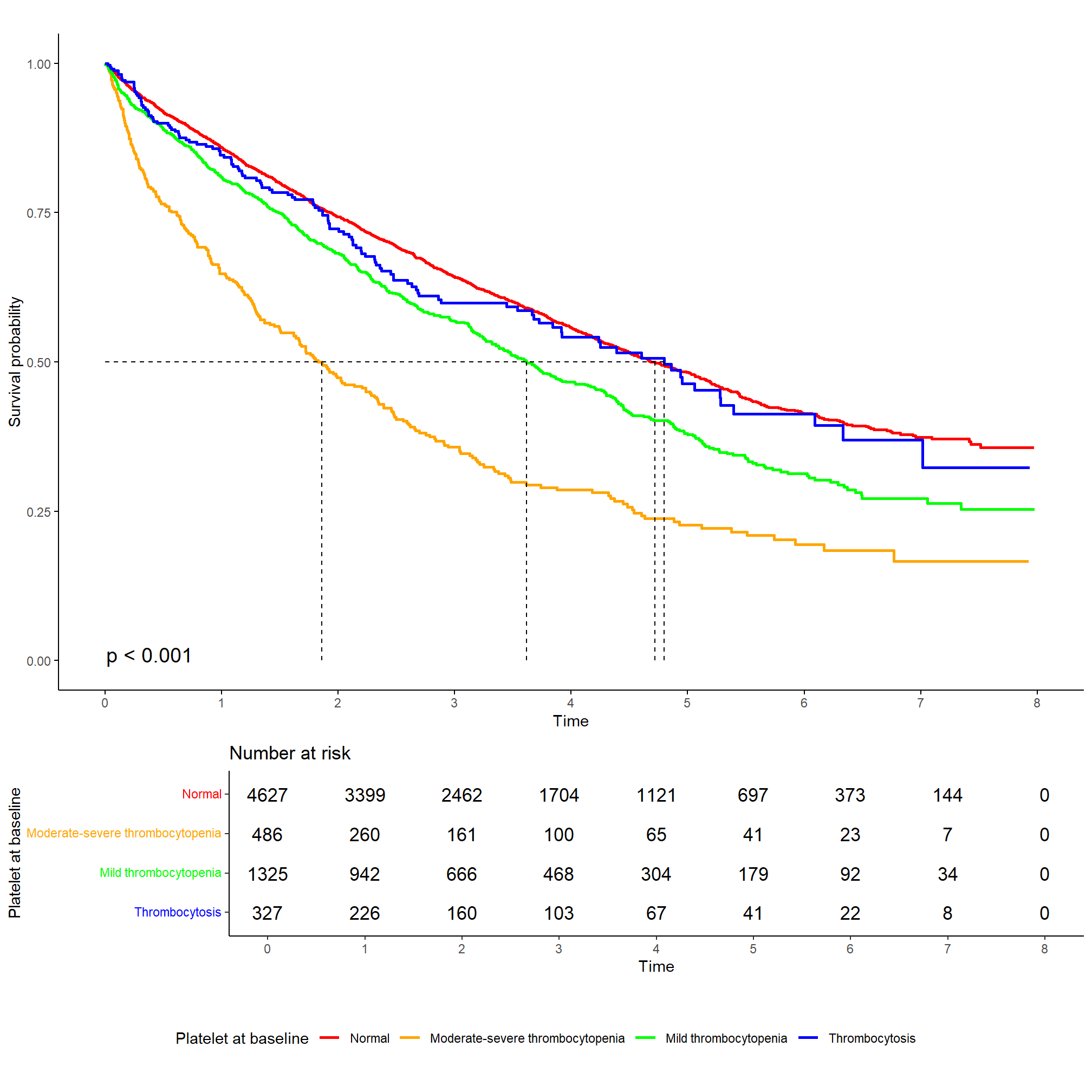


Supplemental Figure 6. OS based on baseline platelet count at MM diagnosis and RVd induction therapy for patients diagnosed ≥2012.


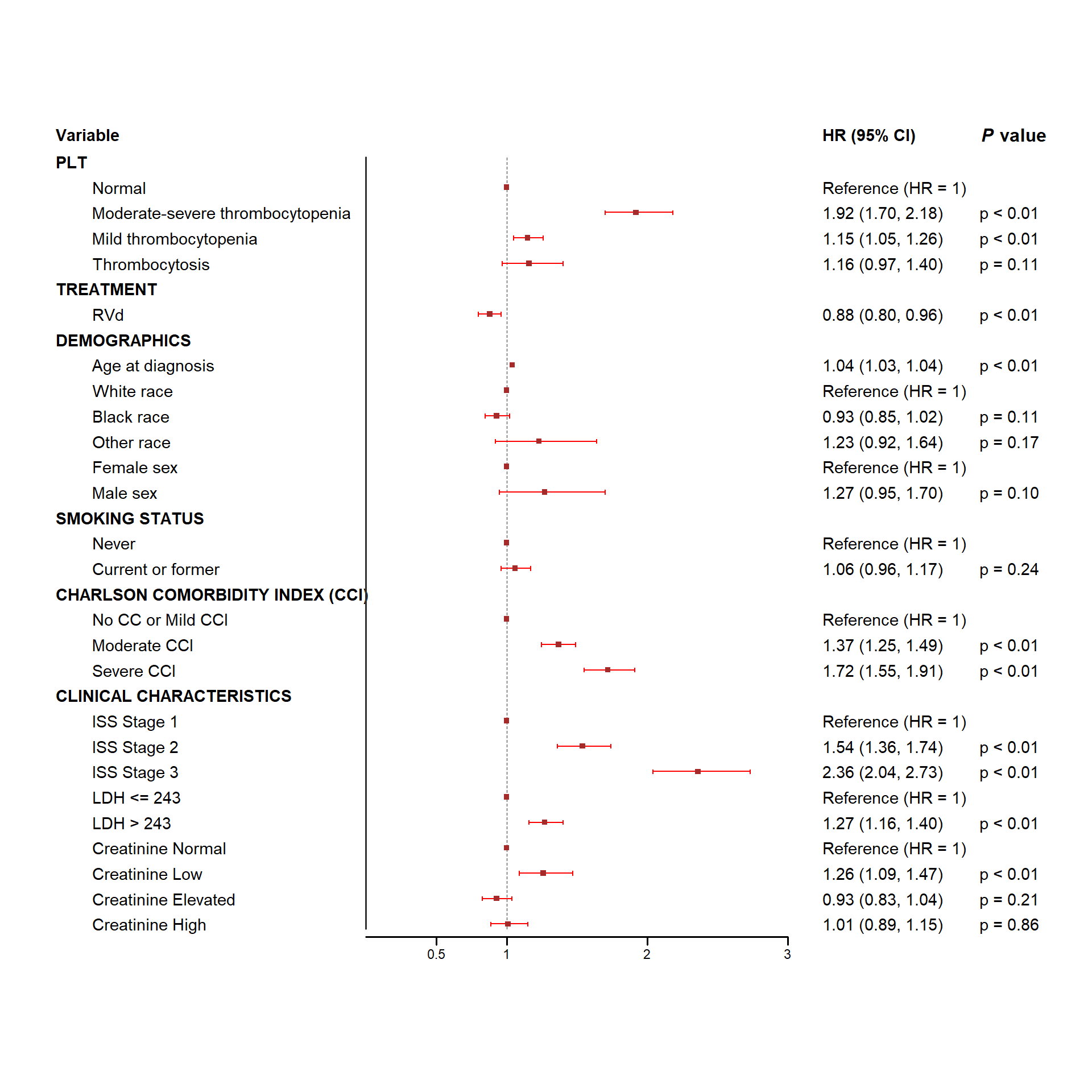


Supplemental Figure 7. OS based on baseline platelet count as both a fixed baseline (up to year 2) and a time-varying covariate (years 2-4).


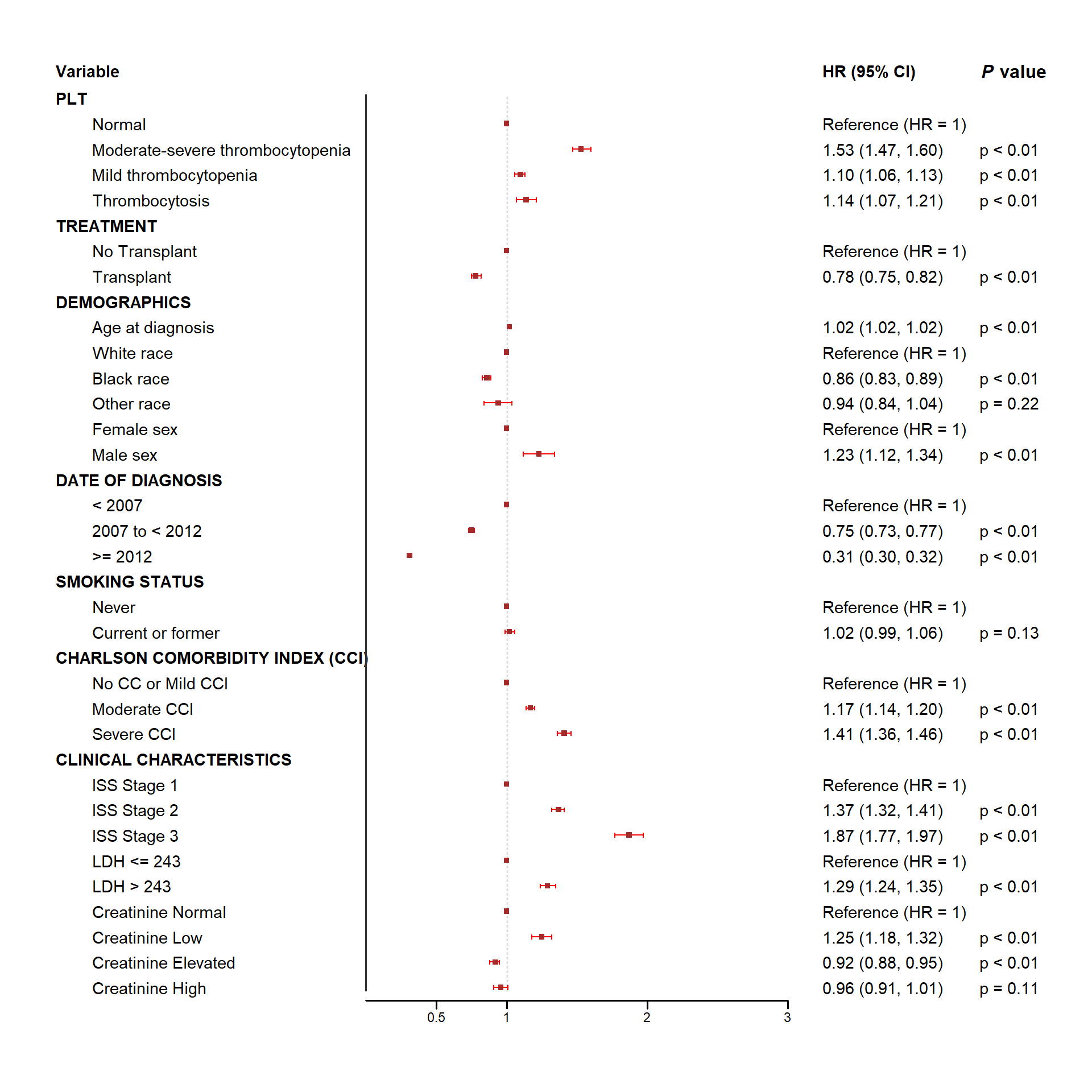

Supplement: Supplementary file 1 — Figure S1: Moderate‐severe thrombocytopenia at baseline, KMs by Platelets at FUP.Figure S2: Mild Thrombocytopenia at baseline, KMs by Platelets at FUP.Figure S3: KM‐Normal Platelets at baseline, KMs by Platelets at FUP.Figure S4: Thrombocytosis at baseline, KMs by Platelets at FUP.Figure S5: OS based on baseline platelet count at MM diagnosis with respect to the era of diagnosis and treatment: (A) <2012 and (B) ≥2012.Figure S6: OS based on baseline platelet count at MM diagnosis and RVd induction therapy for patients diagnosed ≥2012.Figure S7: OS based on baseline platelet count as both a fixed baseline (up to year 2) and a time‐varying covariate (years 2–4). [file JHA2-6-e70153-s001.docx]
